# Alumina-Doped Zirconia Submicro-Particles: Synthesis, Thermal Stability, and Microstructural Characterization

**DOI:** 10.3390/ma12182856

**Published:** 2019-09-05

**Authors:** Gregor Thomas Dahl, Sebastian Döring, Tobias Krekeler, Rolf Janssen, Martin Ritter, Horst Weller, Tobias Vossmeyer

**Affiliations:** 1 Institute of Physical Chemistry, University of Hamburg, Grindelallee 117, 20146 Hamburg, Germany; 2 Electron Microscopy Unit, Hamburg University of Technology, Eißendorfer Straße 42 (M), 21073 Hamburg, Germany; 3 Institute of Advanced Ceramics, Hamburg University of Technology, Denickestraße 15 (K), 21073 Hamburg, Germany; 4 Fraunhofer Center for Applied Nanotechnology CAN, Grindelallee 117, 20146 Hamburg, Germany

**Keywords:** ceramic microparticles, alumina/zirconia, doping, sol-gel, thermal stability, phase transformation, grain growth

## Abstract

Zirconia nanoceramics are interesting materials for numerous high-temperature applications. Because their beneficial properties are mainly governed by the crystal and microstructure, it is essential to understand and control these features. The use of co-stabilizing agents in the sol-gel synthesis of zirconia submicro-particles should provide an effective tool for adjusting the particles’ size and shape. Furthermore, alumina-doping is expected to enhance the particles’ size and shape persistence at high temperatures, similar to what is observed in corresponding bulk ceramics. Dispersed alumina should inhibit grain growth by forming diffusion barriers, additionally impeding the martensitic phase transformation in zirconia grains. Here, alumina-doped zirconia particles with sphere-like shape and average diameters of ∼300 nm were synthesized using a modified sol-gel route employing icosanoic acid and hydroxypropyl cellulose as stabilizing agents. The particles were annealed at temperatures between 800 and 1200 ${}^{\circ }C$ and characterized by electron microscopy, elemental analysis, and X-ray diffraction. Complementary elemental analyses confirmed the precise control over the alumina content (0–50 mol%) in the final product. Annealed alumina-doped particles showed more pronounced shape persistence after annealing at 1000 ${}^{\circ }C$ than undoped particles. Quantitative phase analyses revealed an increased stabilization of the tetragonal/cubic zirconia phase and a reduced grain growth with increasing alumina content. Elemental mapping indicated pronounced alumina segregation near the grain boundaries during annealing.

## 1. Introduction

Micro- and nanoscale zirconia ceramics and zirconia-based composite materials have attracted significant scientific interest due to their very favorable properties, such as exceptional thermal stability, wear resistance, fracture toughness, high refractive index, chemical inertness, thermal insulation, and bio-compatibility [1,2]. These characteristics give rise to a number of potential applications, including corrosion protection [3], catalysis [4], chromatography [5], sensors [6,7], and solid oxide fuel cells [8,9], as well as bioceramics [10,11,12].

**Figure 1:**
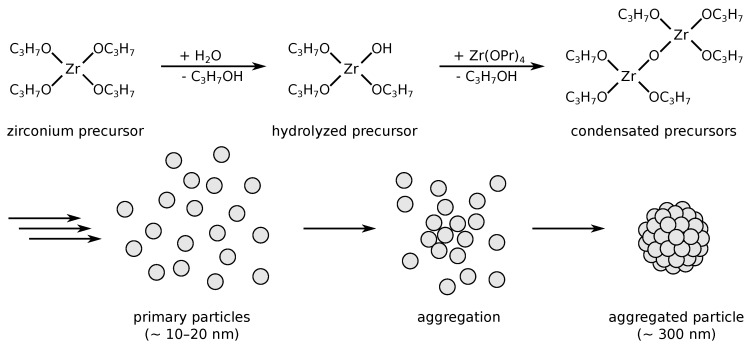
Schematic representation of aggregation-based synthesis of uniform, porous sol-gel zirconia submicro-particles from zirconium *n*-propoxide. Hydrolysis and condensation of the precursors is followed by primary particle formation and subsequent aggregation. Doped particles are obtained analogously by the use of mixed precursors.

Furthermore, recent advances in the wet-chemical synthesis of uniformly shaped ceramic particles have enabled their use as building-blocks in functional optical materials, i.e., photonic crystals or glasses, for potential applications as thermophotovoltaic (TPV) absorbers/emitters, as well as structural coloration, and advanced thermal barrier coatings (TBCs) [13,14,15]. For these applications, spherical particles with sizes in the micron and submicron ranges are required in order to manipulate the materials’ interaction with light within the infrared and visible ranges. Thus, the size and shape control of particles during their synthesis is of major importance for tuning the optical properties of such photonic coatings. Spherical zirconia particles in the nano- and micrometer size regime have become accessible by various approaches. Among others, the sol-gel route appears particularly promising due to its simplicity, the moderate process temperatures and the low-cost equipment needs [1]. Here, several studies in the recent past have contributed to a deeper understanding of the processes involved in particle formation and growth during a sol-gel synthesis and the impact of various reaction parameters. The early foundations of this progress have been laid by Fegley et al., who reported the synthesis of monodispersed ZrO_2_ powders by the controlled hydrolysis and polycondensation of zirconia alkoxides in an ethanolic solution [16]. Ogihara et al. subsequently proposed a protocol for the fabrication of monosized spherical zirconia particles by a related method with focus on the control over shape uniformity, size distribution and state of agglomeration, followed by another study on the particle growth mechanism [17,18]. In agreement with other reports, a particle growth based on the aggregation of smaller primary particles with diameters of 10 to 20 nm is assumed, as schematically illustrated in Figure 1, rather than a conventional LaMer-type particle growth [17,18,19,20]. Further improvements of this method were achieved by Lerot et al., Yan et al., and Widoniak et al. [20,21,22], by the addition of fatty acids, polymers, or salts as stabilizing agents to gain more precise control over particle size and size distribution. In a more recent work, our group demonstrated the controlled synthesis of monosized and uniform zirconia particles with diameters between 0.4 and 4.3 μm, based on some of these previous protocols. We also studied the effect of temperatures up to 1500 ${}^{\circ }C$ on the structural stability and morphology of these particles [23].

The stability of the particles under thermal stress is particularly important for optical high-temperature applications, where the functionality is based on the material’s well-defined shape and structure. As in the bulk material, the high-temperature performance of zirconia particles can be improved by doping with foreign metal oxides, predominantly yttria, but also magnesia, calcia, ceria, scandia, samaria and others [1,23,24,25]. The stabilizing effect is attributed to the inhibition of the destructive martensitic phase transformation from tetragonal (t) to monoclinic (m) zirconia and the inhibited grain growth caused by the solute drag effect [26,27]. In addition, the t→m transformation can be used to enhance fracture toughness and strength [2,28]. Furthermore, zirconia–alumina composites have been linked to enhanced thermomechanical properties as well as toughness and strength [29,30,31,32,33,34,35]. However, several studies on the ZrO_2_/Al_2_O_3_ system published in the recent past concentrate on dense ceramics or heterogeneous powders and do not allow for precise predictions regarding the properties of porous and monosized spherical submicro-particles fabricated by sol-gel synthesis [36,37,38]. To the best of our knowledge, there has been no systematic study on the synthesis and high-temperature performance of alumina-doped zirconia submicro-particles fabricated by sol-gel synthesis, to date.

The aim of this work was the fabrication of alumina-doped zirconia submicro-particles by sol-gel synthesis and the examination of their high-temperature performance. For this purpose, zirconia particles with varying alumina content were synthesized with the aid of co-stabilizing agents. The impact of the alumina/zirconia molar ratio on the formation and stability of crystal phases and corresponding grain sizes after exposure to thermal stress was investigated. As-prepared particles were characterized using (scanning) transmission electron microscopy((S)TEM), scanning electron microscopy (SEM), energy-dispersive X-ray spectroscopy (EDX), inductively-coupled plasma optical emission spectrometry (ICP-OES) and powder X-ray diffraction spectrometry (XRD), before and after annealing at temperatures between 800 and 1200 ${}^{\circ }C$.

## 2. Results and Discussion

**Figure 2:**
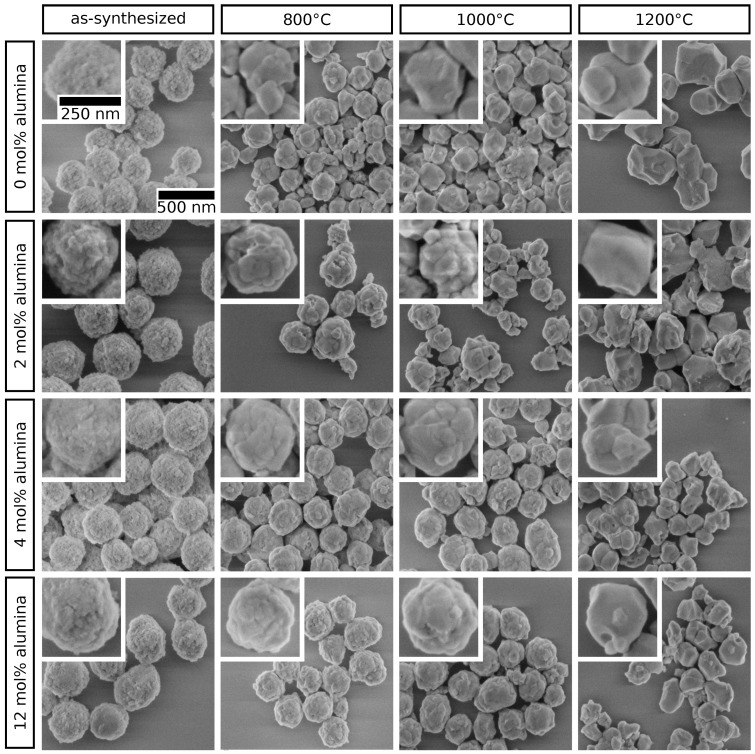
Representative SEM micrographs of zirconia particles doped with 0, 2, 4 and 12 mol% alumina, as-synthesized and after annealing at 800, 1000 and 1200 ${}^{\circ }C$ for 3 h.

In a previous study we reported a protocol for the synthesis of yttria-doped spherical zirconia microparticles via a facile sol-gel route [23]. In our present work, this method was adapted to produce alumina-doped zirconia submicro-particles with average diameters of approximately 300 nm (as-synthesized, before annealing) using zirconium *n*-propoxide and aluminum *iso*-propoxide as precursors. This particular particle size was chosen because zirconia particles in the low submicron size regime are considered interesting building blocks for optical applications, such as structural coloration [39]. The alumina content *x* ((AlO_1.5_)_x_(ZrO_2_)_1−x_) was varied systematically between 0 and 50 mol% by adjusting the ratio of the precursors in the reaction mixture. Furthermore, an organic stabilizer mixture of icosanoic acid and hydroxypropyl cellulose (HPC) was used for size and shape control of the particles. Thereby, HPC, which is commonly used in nanomaterials synthesis, was employed to enable the desired particle sizes in the low submicron regime [40]. Moreover, it has been suggested that HPC minimizes particle agglomeration [21]. In first, preliminary experiments it was observed that the particle diameter decreased with increasing alumina content. It has also been shown previously that the thermal stability of such porous sol-gel zirconia particles depends strongly on the particle size [23]. Therefore, in order to meet the requirement of comparable particle diameters for all samples, the particle sizes were individually adjusted by varying the organic stabilizer composition in the solution during synthesis. Small changes of the organic stabilizer ratio were sufficient for a significant yet controllable impact on the resulting particle size. This adjustment enabled the fabrication of samples with very similar mean diameters of ∼300 nm but very different alumina doping levels. Details are provided in the Supplementary Material (Tables S1 and S2). Representative SEM micrographs of selected as-synthesized samples are shown in Figure 2 (left column).

**Figure 3:**
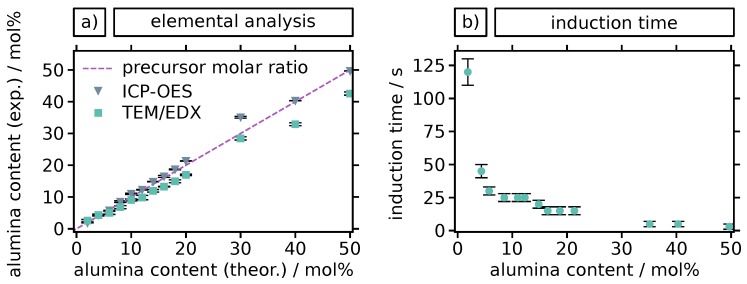
(**a**) Elemental analysis of zirconia particles with varying (theoretical) alumina content based on the molar composition of precursors. Zr and Al elemental fractions were quantified by TEM/EDX and ICP-OES. EDX results are based on two to four individual particles per sample, OES results are averaged over a large number of particles. Error bars indicate the standard deviation of multiple measurements. (**b**) Induction times measured during the syntheses of particles with varying alumina content (as determined by ICP-OES). The induction time decreases strongly with increasing alumina content in the range of 2 to 8 mol%. Error bars indicate the estimated uncertainty of the determination of the end of induction.

The alumina content of each particle sample was quantified using TEM/EDX and ICP-OES analysis. Respective findings are shown in Figure 3a. The data of both analytical methods are in quite good agreement with the molar ratios of precursors adjusted in the reaction mixture. While ICP-OES provides very accurate values for the average Al elemental content of the samples, it cannot provide data regarding the composition of individual particles. In contrast, the EDX data were recorded on the single particle level. Thus, the rather good agreement of both data sets confirmed that the alumina content of the individual particles corresponds very well to the average alumina content of the samples, and that the doping level could easily be controlled by adjusting the precursor ratio of the reaction mixture.

Additionally, induction times, i.e., the time between the initiation of the reaction and the visible change of the solution from transparent to white, were measured during all syntheses. They are shown in Figure 3b. A clear trend of decreasing induction time with increasing alumina content is observed. In sol-gel procedures, the induction time is a suggestive quantity that provides information about the rate of hydrolysis. It is commonly argued that short induction times indicate fast hydrolysis and vice versa [18,20,21,22]. Interestingly, here the induction time decreased significantly with increasing alumina content, particularly at low alumina contents. This implies a faster hydrolysis with a higher percentage of Al-precursor in the mixture. It has been suggested for similar syntheses of yittria-doped zirconia particles, that trivalent alkoxide precursors have a higher hydrolysis rate due to a reduced sterical shielding of the metal center, compared to tetravalent alkoxides, e.g., Zr-propoxide [41]. Additionally, aluminum is a hard acid according to the HSAB concept (hard/soft acids/bases) and therefore more likely than zirconium to accept electrons from the hard oxygen during the hydrolysis.

### 2.1. Thermal Stability of Alumina-Doped Zirconia Particles

In order to investigate the impact of high temperatures on the morphology and structural integrity of the alumina-doped particles, all samples were annealed at 800, 1000 and 1200 ${}^{\circ }C$ for 3 h, excluding heating and cooling ramps. It has been observed previously, that zirconia particles prepared via the sol-gel approach are initially porous and show significant densification, accompanied by a decrease in size upon annealing [23]. The subsequent characterization was performed by TEM, SEM and powder XRD. Representative SEM images of selected samples are depicted in Figure 2. It is clearly visible that the alumina content has a significant effect on the structural integrity of the particles after heat treatment. The undoped particles are partly disintegrated after heating to 800 ${}^{\circ }C$, with a significant increase in grain sizes, and strongly fractured and faceted morphology after heating to 1000 and 1200 ${}^{\circ }C$. Particles with an alumina content of 2 mol% show improved stability against disintegration after annealing at 800 ${}^{\circ }C$ and smaller grains than the undoped sample. After annealing at 1000 ${}^{\circ }C$ however, the particles are predominantly disintegrated. For the particle sample with 4 mol% alumina a significant improvement in structural integrity after annealing at 800 and 1000 ${}^{\circ }C$ is observed. Fracturing and grain coarsening is strongly inhibited in contrast to the undoped sample. A similar behavior is observed for the particles with an alumina content of 12 mol% or higher. Still after annealing at 1200 ${}^{\circ }C$ all particles were disintegrated, independent of their alumina content.

### 2.2. Crystal Phase and Microstructure Analysis

**Figure 4:**
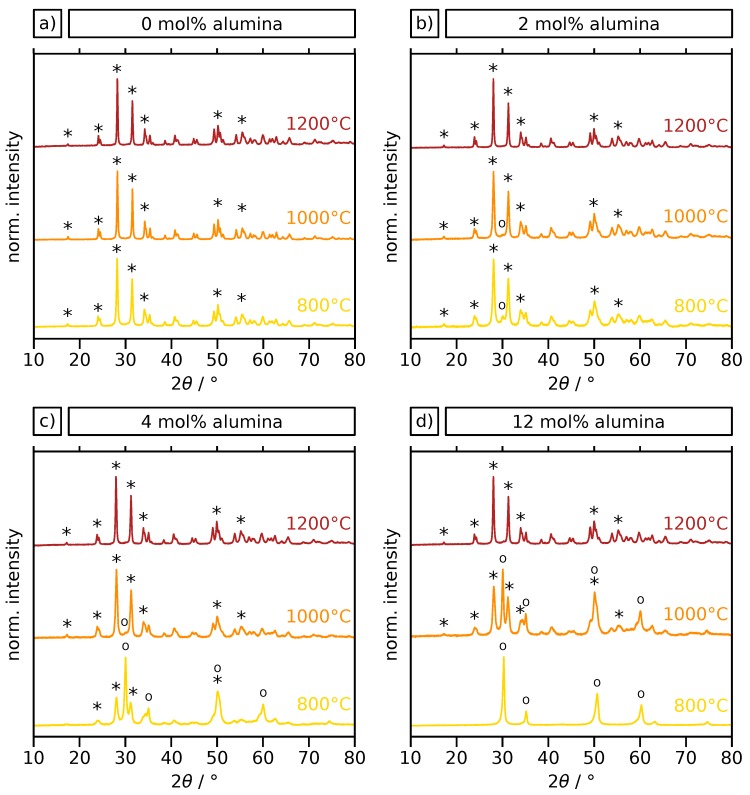
XRD diffractograms of zirconia particles doped with (**a**) 0, (**b**) 2, (**c**) 4 and (**d**) 12 mol% alumina, after annealing at 800, 1000 and 1200 ${}^{\circ }C$ for 3 h. Selected peaks of monoclinic (*) and tetragonal/cubic (∘) zirconia are indicated accordingly.

XRD powder diffractograms for the selected particle samples after annealing are depicted in Figure 4. A full set of diffractograms is provided in the Supplementary Material (Figure S4). Characteristic peaks are indicated for the tetragonal/cubic and monoclinic zirconia phases. It is clearly notable that the undoped sample is fully crystallized in the monoclinic phase (a) after annealing at 800 and 1000 ${}^{\circ }C$, while the tetragonal/cubic phase is increasingly stabilized when alumina-doping is increased (b,c,d). Furthermore, significant line broadening with increasing alumina content indicates a decrease in crystallite sizes. As already discussed, crystal phase transformations and grain growth are the most relevant mechanisms for the destabilization of zirconia ceramics. In order to examine the microstructure of the particle samples, the XRD data was quantitatively evaluated by Rietveld refinement. Crystalline alumina phases were not found for any of the investigated samples, although all known alumina polymorphs are expected to crystallize at temperatures lower than 1100 ${}^{\circ }C$ [42]. It indicates the presence of very fine-grained dispersed alumina-phase, because such small-scale crystallites are difficult to detect in XRD due to pronounced line broadening, especially when only present as a minor phase. A glassy, non-crystalline alumina phase is conceivable, too, assuming the crystallization is deferred towards higher temperatures as a consequence of the presence of zirconia and the structural constraint. This is demonstrated exemplarily for a sample with 20 mol% alumina, annealed at 1500 ${}^{\circ }C$ in the Supplementary Material (Figure S5). These observations are in good agreement with previous findings for comparable materials fabricated by chemical vapor deposition [37].

**Figure 5:**
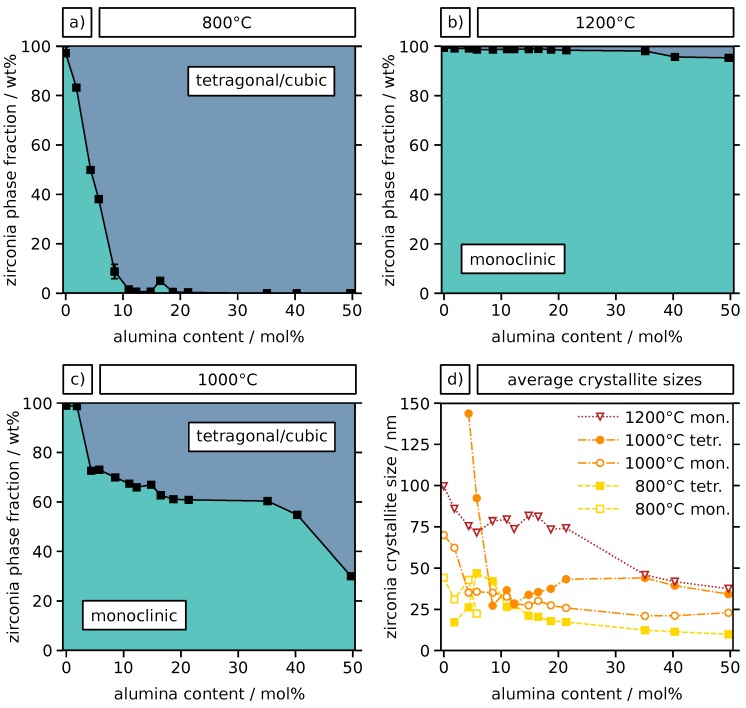
Quantitative phase analysis and crystallite sizes of Al-doped zirconia particles with different Al content after annealing at (**a**) 800 ${}^{\circ }C$, (**b**) 1000 ${}^{\circ }C$ and (**c**) 1200 ${}^{\circ }C$ for 3 h, from XRD data using Rietveld refinement. The phase compositions are shown as weight fractions of the zirconia tetragonal/cubic and monoclinic phases. (**d**) Monoclinic and tetragonal crystallite sizes were obtained from the same data and are only shown for crystal phases with a minimum weight fraction of 10%.

In the Rietveld analysis, cubic zirconia needed to be included in the tetragonal fraction, as they are difficult to distinguish when distinct line broadening occurs. The obtained phase compositions for the investigated temperatures are presented in Figure 5. The data clearly show that the tetragonal/cubic phase is increasingly stabilized with increasing alumina content after annealing at 800 ${}^{\circ }C$ (a), as suggested by the XRD patterns in Figure 4. A nearly complete stabilization is reached at an alumina content of 10 mol%. After annealing at 1000 ${}^{\circ }C$ (b) a similar trend is observed with 70% tetragonal/cubic phase for the highest alumina content of 50 mol%. However, after this temperature treatment, complete stabilization of the tetragonal/cubic polymorph is not achieved for any of the particle samples. After annealing at 1200 ${}^{\circ }C$ (c) almost no tetragonal/cubic fraction is detected. Only for the higher Al contents of 40 mol% and 50 mol% a small tetragonal share of ∼5% is present. Obviously, the addition of alumina inhibits the destructive t→m phase transformation in the zirconia, which prevents mechanical stress and ultimately contributes to an increased structural stability during annealing. While such a stabilization is commonly partly attributed to a favorable lattice distortion in yttria- or ceria-stabilized zirconia, for alumina-stabilized zirconia, this is mostly attributed to an inhibited grain growth due to constraint [30,41]. Phase diagrams reported in previous studies on the zirconia–alumina system suggest that the crystal phase of zirconia is expected to be monoclinic up to 1150 ${}^{\circ }C$, mostly independent of the alumina content [43]. In contrast, the results in this study show a significant impact of the alumina content on the zirconia crystal phase. We attribute this finding to the fact that phase diagrams represent the equilibrium at usually much larger grain sizes than present in our sol-gel derived microparticles with nanocrystalline and porous microstructure. Furthermore, our samples were annealed for 3 h and are unlikely to have reached an equilibrium state. In order to clarify the reason for the differences in stabilization of the tetragonal/cubic zirconia phase, average zirconia crystallite sizes were extracted from the Rietveld data for all samples annealed at 800, 1000 and 1200 ${}^{\circ }C$. This analysis allows for determining the crystallite sizes of the tetragonal/cubic and monoclinic zirconia phases separately. The results are presented in Figure 5d, showing only data for phases with a minimum weight fraction of 10%.

As expected, particles annealed at higher temperatures generally show a larger average crystallite size than those annealed at lower temperatures due to enhanced grain growth. For all temperatures, an overall decrease in crystallite size is observed with increasing alumina content. A possible explanation could be given using the solute drag effect. However, although the lower valence of Al compared to Zr does enable this mechanism, a strong solute drag is commonly attributed to dopants with different valence and higher ionic radius. The latter is not the case for Al, therefore a different mechanism is considered more likely. Segregation occurs during sintering virtually forming barriers of alumina at the grain interfaces and consequently pinning the grain boundaries [26,44,45]. The mass transport of zirconia along the grain boundaries that is required to enable grain growth is likely to be additionally inhibited compared to dense materials due to the porous and agglomerate nature of the particles. Interestingly, at low (<8 mol%) and again at higher alumina content (>20 mol%) the tetragonal/cubic crystallites are larger than the monoclinic ones for the sample annealed at 1000 ${}^{\circ }C$ (Figure 5d). This observation is contradictory to the expectation, i.e., smaller grains are preferably tetragonal and larger grains monoclinic, based on earlier studies [46]. However, similar observations were made earlier concerning La-doped zirconia microparticles [47]. Here, it was suggested that the formation of smaller monoclinic than tetragonal grains is due to either twinning during the phase transformation or nucleation at the dopant-rich grain boundaries.

**Figure 6:**
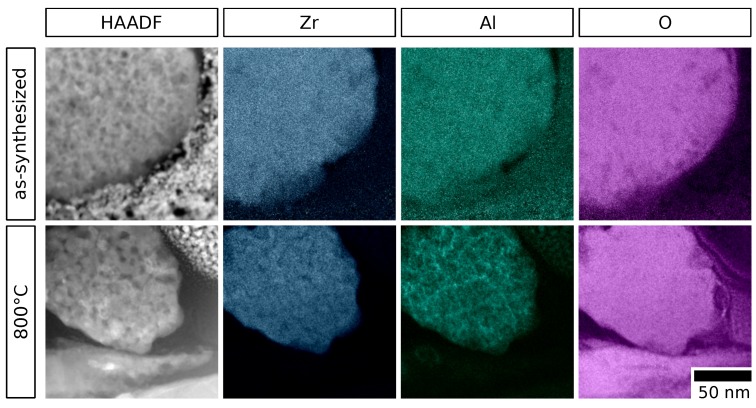
Elemental maps recorded from FIB lamellae of single particles with 20 mol% Al content, as-synthesized (**top**) and after annealing at 800 ${}^{\circ }C$ for 3 h (**bottom**). Corresponding HAADF images are shown along with the elemental distributions of Zr, Al and O. The particles clearly show a homogeneous elemental spatial distribution after synthesis and strong segregation after the annealing.

For an as-synthesized and a selected annealed particle sample (800 ${}^{\circ }C$) with 20 mol% alumina content, lamellae with thicknesses of approximately 100 nm were prepared by focused ion beam technique. These lamellae were analyzed using EDX elemental mapping in combination with STEM. The obtained maps and the corresponding high-angle annular dark-field (HAADF) micrographs are depicted in Figure 6. They clearly show a homogeneous distribution of Zr, Al and O across the entire particle before annealing. In contrast, after annealing at 800 ${}^{\circ }C$, alumina segregation is observed in a network-like structure, in rather Zr-lean areas. In contrast, oxygen is distributed homogeneously, disproving a general accumulation due to porosity. In comparison to the HAADF image of the annealed particle, it is apparent that alumina accumulates near the grain boundaries. This dopant segregation is in agreement with similar observations in different mixed Zr/Al oxides and other doped zirconia materials [37,48]. This finding supports the assumption that segregated inter-grainar alumina is substantially involved in the observed grain growth inhibition. A significant pinning of the grain boundaries due to segregated dopant is likely, as also suggested previously [37,44].

## 3. Materials and Methods

### 3.1. Materials

1-propanol (99.9%, anhydrous) and zirconium(IV) *n*-propoxide (70 wt% in *n*-propanol) were purchased from Alfa Aesar (Kandel, Germany). Aluminum(III) *iso*-propoxide (99.99%) was ordered from ABCR (Zürich, Switzerland). Ethanol absolute (99.5%, extra dry) was from ACROS Organics (Geel, Belgium) and 1-butanol (99.5%) was purchased from TH.GEYER (Renningen, Germany). Icosanoic acid (99.0%), hydroxypropyl cellulose (99%, M_w_ = 80000 g mol^−1^) and demineralized water (ACS reagent) were obtained from Sigma Aldrich (Munich, Germany).

### 3.2. Particle Syntheses

Zirconia microparticles were fabricated by a modified sol-gel protocol based on the works of Widoniak et al. and Leib et al. in an inert gas atmosphere [20,23]. Varying amounts of icosanoic acid and hydroxypropyl cellulose were dissolved in 45 mL ethanol absolute and heated to 55 ${}^{\circ }C$ under constant stirring. A precursor suspension was prepared by mixing zirconium *n*-propoxide solution and aluminum *iso*-propoxide in the required molar ratio and adding 5 to 8 mL 1-propanol in a nitrogen atmosphere. The sealed mixture was ultrasonicated for 10 to 20 min until complete dispersion of the solid. 190 μL demineralized water were added to the stabilizer solution and after 15 min the precursor suspension was injected quickly. After visible inset of turbidity (end of induction time), the stirring speed was reduced and the mixture was aged for 3 h at 55 ${}^{\circ }C$. It was subsequently transferred into ice cold 1-butanol and purified by repeated centrifugation (10000 RCF, 4 min, 4 ${}^{\circ }C$), decanting of the supernatant and washing with 25 mL ethanol. This purification was conducted 3 times with decreasing centrifugation speed down to 6000 RCF, finally. The purified particles were resuspended in 10 mL ethanol. A full list of reagent quantities and observed induction times used for all particle syntheses is provided in the supporting information. For the undoped sample a different batch of the precursor reagent was used causing a significant deviation in induction time and stabilizer adjustment requirement. The respective data set was therefore excluded from the evaluation of the induction times.

### 3.3. Annealing Experiments

Prior to high-temperature annealings, all particle samples were dried at ambient conditions overnight and further dried at 80 ${}^{\circ }C$ for 4 h. The samples were ground using an agate mortar. An amount of 30 mg of each sample was used per annealing experiment and heated to either of the target temperatures (800, 1000 and 1200 ${}^{\circ }C$) at a heating rate of 5 ${}^{\circ }C$ min^−1^. The target temperature was kept for 3 h. Afterwards, the samples were allowed to cool down to ambient temperature at cooling rates of ≤5 ${}^{\circ }C$ min^−1^. The annealed particle samples were ground using an agate mortar and resuspended in ethanol by ultrasonication for 10 to 20 min for further characterization.

### 3.4. Characterization

TEM measurements were conducted using a JEM-1011 HRTEM instrument (JEOL, Tokyo, Japan) operating at 100 kV. EDX measurements were carried out with a HT-TEM CM 300 (Philips, Hamburg, Germany) operating at 200 kV. For TEM/EDX sample preparation, a volume of 10 μL of the readily mixed particle suspension was drop-casted onto a carbon coated copper grid and left to dry for at least 12 h. Mean particle sizes were determined by evaluation of TEM images using the software ImageJ 1.51p. For this, the average particle diameters were calculated from the obtained projections using the area of a circle, assuming perfectly circular particles. SEM characterization was performed using a GEMINI LEO 1550 HRSEM (Carl Zeiss, Oberkochen, Germany) operating at 2 kV. A complete set of TEM micrographs is provided in the Supplementary Materials (Figures S1–S3).

ICP-OES measurements were done using a SPECTRO ACROS spectrometer (SPECTRO Analytical Instruments, Kleve, Germany) with a plasma power of 1.40 kW. For ICP-OES sample preparation an optimized carbonate fusion protocol was used [49]. A portion of 10 mg of each dried particle sample was used per measurement and at least a double determination was conducted for every sample and the results were averaged. Detailed characterization results are provided in the Supplementary Materials (Table S2).

Powder XRD diffraction measurements were performed using a PANalytical X’Pert PRO MPD instrument (Philips, Hamburg, Germany) with a Cu anode, measuring a 2θ range between 10 ${}^{\circ }$ and 80 ${}^{\circ }$ at a step size of 0.033 ${}^{\circ }$ and a time per step of 75 s. For XRD sample preparation 200 μL of the readily mixed particle suspension was drop-casted into a PTFE ring fixed onto a silicon single crystal (911) mount and left to dry for at least 24 h. The obtained diffractograms were background corrected and evaluated using the software Maud 2.76 and its built-in Rietveld refinement tool [50]. For this, an instrument calibration was conducted prior to the sample measurements in order to account for instrumental peak broadening and other instrumental characteristics. Crystallographic information for all zirconia phases originated from a work by Howard et al. and were obtained from the ICSD online database (ICSD codes: 62993, 62994, 62995) [51]. Crystallographic data for α-alumina was obtained from a study by Lutterotti et al. and retrieved from the COD online database (COD code: 1000032) [50]. Detailed data of the refinement results and a full set of Rietveld graphs are provided in the Supplementary Materials (Table S3, Figures S6–S9).

Thin lamellae (thickness ≤ 100 nm) of particle samples were prepared by platinum deposition on drop-casted particle samples and focused Ga^+^ ion beam thinning using a Helios G3 UC (FEI, Hillsboro, OR, USA). EDX elemental maps and STEM/HAADF images were acquired using a Talos F200X (FEI, Hillsboro, OR, USA) equipped with Super-X EDS, operating at 200 kV. EDX signals were detected in a range of 0 to 20 keV and the lines at 1.4875 keV (Al K${}_{\alpha 1}$) and 15.7749 keV (Zr K${}_{\alpha 1}$) were used for quantification assuming no absorption and applying a Brown Powell ionization cross-section model in the software Velox (FEI, Hillsboro, OR, USA).

## 4. Summary and Conclusions

Alumina-doped zirconia particles with sphere-like shape and average diameters in the submicron range were synthesized using a sol-gel approach, assisted by the use of icosanoic acid and hydroxypropyl cellulose as stabilizing agents. The alumina content varied between 0 and 50 mol% by adjusting the molar ratio of the used Zr- and Al-alkoxide precursors, while the stabilizer composition needed to be tuned for each sample in order to maintain a comparable particle size of ∼300 nm. The particle characterization demonstrated a precise control over particle size, shape and alumina content.

The thermal stability of the particles was investigated by annealing the dried particle samples at temperatures between 800 and 1200 ${}^{\circ }C$. Electron microscopy images revealed a significantly increased thermal stability of alumina-doped particles compared to undoped particles. X-ray diffraction results showed the tetragonal/cubic zirconia phases to be increasingly stabilized by alumina-doping. Further, the crystallite sizes determined by Rietveld analysis indicated a reduced grain size with increasing alumina content. EDX mapping revealed significant alumina segregation at the grain boundaries after annealing. These results were in good agreement with previous findings in comparable bulk systems, supporting the hypothesis of material strengthening by alumina segregation and grain boundary pinning [29,37]. Therefore, we conclude that alumina-doping of sol-gel-derived zirconia microparticles provides a well-controllable tool for the precise modification of the particle material and the enhancement of their structural stability at high temperatures. In order to gain further insight into the underlying mechanisms of stabilization, subsequent more in-depth experiments are desirable, e.g., in situ diffraction and micro-compression measurements, as reported for related particle systems [52]. Furthermore, this work can lay the foundation for research on more complex material systems, such as ternary or quaternary oxide particles. The particles fabricated and characterized in this work can be considered for a number of potential uses, especially as building blocks for functional optical materials in high-temperature applications.

## Supplementary Materials

The following are available online at https://www.mdpi.com/1996-1944/12/18/2856/s1, Table S1. Reaction conditions for all particle syntheses, Figure S1. Representative TEM micrographs of zirconia particles doped with 0, 2, 4, 6, and 8 mol% alumina, as synthesized and after annealing at 800, 1000 and 1200 ${}^{\circ }C$, Figure S2. Representative TEM micrographs of zirconia particles doped with 10, 12, 14, 16, and 18 mol% alumina, as synthesized and after annealing at 800, 1000 and 1200 ${}^{\circ }C$, Figure S3. Representative TEM micrographs of zirconia particles doped with 20, 30, 40, and 50 mol% alumina, as synthesized and after annealing at 800, 1000 and 1200 ${}^{\circ }C$, Table S2. TEM, EDX, and ICP-OES characterization results for all as-synthesized particle samples, Figure S4. Powder X-ray diffractograms for all particle samples, after annealing at 800, 1000 and 1200 ${}^{\circ }C$ for 3 h, Figure S5. Powder X-ray diffractogram for a particle sample doped with 20 mol% alumina, after annealing at 1500 ${}^{\circ }C$ for 3 h, Table S3. XRD characterization results extracted from Rietveld-refined diffractograms for all particle samples, annealed at 800, 1000 and 1200 ${}^{\circ }C$, Figures S6–S9. Rietveld graphs for all annealed particle samples.
