# Supplementary material for: Alumina-Doped Zirconia Submicro-Particles: Synthesis, Thermal Stability, and Microstructural Characterization
Source: Materials (Basel). 2019 Sep 5;12(18):2856. doi: 10.3390/ma12182856 (PMC6766039; doi:10.3390/ma12182856)
Supplement: Supplementary file 1 [file materials-12-02856-s001.pdf]

Supplementary Materials: Alumina-doped zirconia submicro-particles: synthesis, thermal stability, and microstructural characterization

Gregor Thomas Dahl [1], Sebastian Döring [1,†], Tobias Krekeler [2], Rolf Janssen [3], Martin Ritter [2], Horst Weller [1,4] and Tobias Vossmeyer [1,*]

Table S1. Reaction conditions for all particle syntheses. All reactions were carried out in a total volume of 50 to 53 mL ethanol/1-propanol at 55 °C, under moisture exclusion in a nitrogen atmosphere. Pure Al-*iso*-propoxide and a 70 wt% solution of Zr-*n*-propoxide served as precursors. A different Zr-precursor batch was used for the sample with an alumina content of 0 mol% than for all other samples.

alumina content/mol%	Al-precursor/mg	Zr-precursor/mg	water/µL	induction time/s	HPC/mg	icosanoic acid/mg
0	–	1690	180	25	63.0	26.0
2	15	1650	190	120	56.0	23.4
4	29	1610	190	45	58.4	21.4
6	44	1580	190	30	55.8	23.6
8	59	1540	190	25	56.1	23.2
10	73	1510	190	25	55.9	23.3
12	88	1480	190	25	55.7	23.4
14	104	1440	190	20	55.5	23.6
16	118	1410	190	15	53.7	24.3
18	132	1380	190	15	52.1	25.6
20	148	1340	190	15	52.0	25.1
30	220	1180	190	5	48.4	27.8
40	293	1010	190	5	44.7	30.3
50	366	830	190	3	28.2	40.5

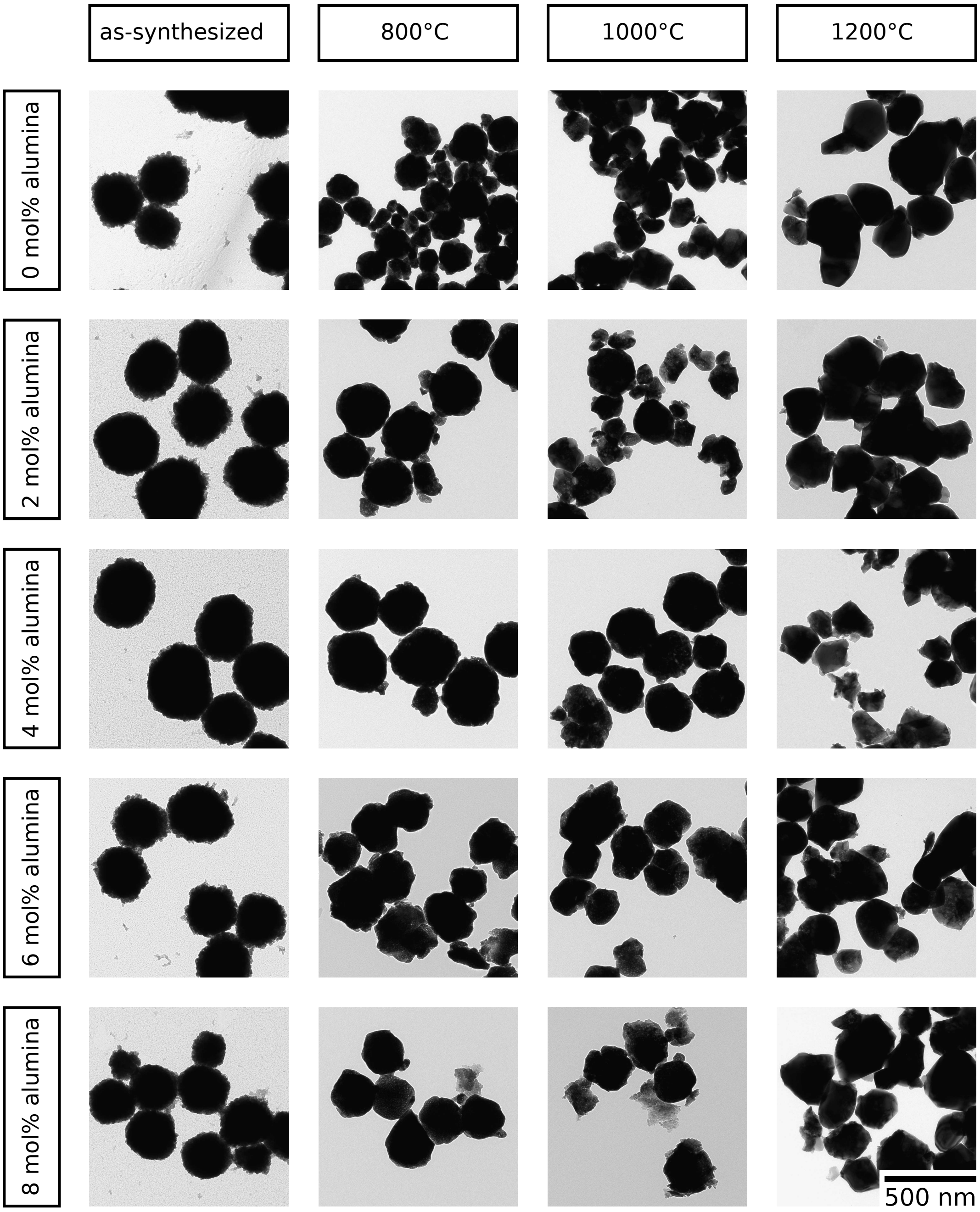

Figure S1. Representative TEM micrographs of zirconia particles doped with 0, 2, 4, 6, and 8 mol% alumina, as synthesized and after annealing at 800, 1000 and 1200 °C.

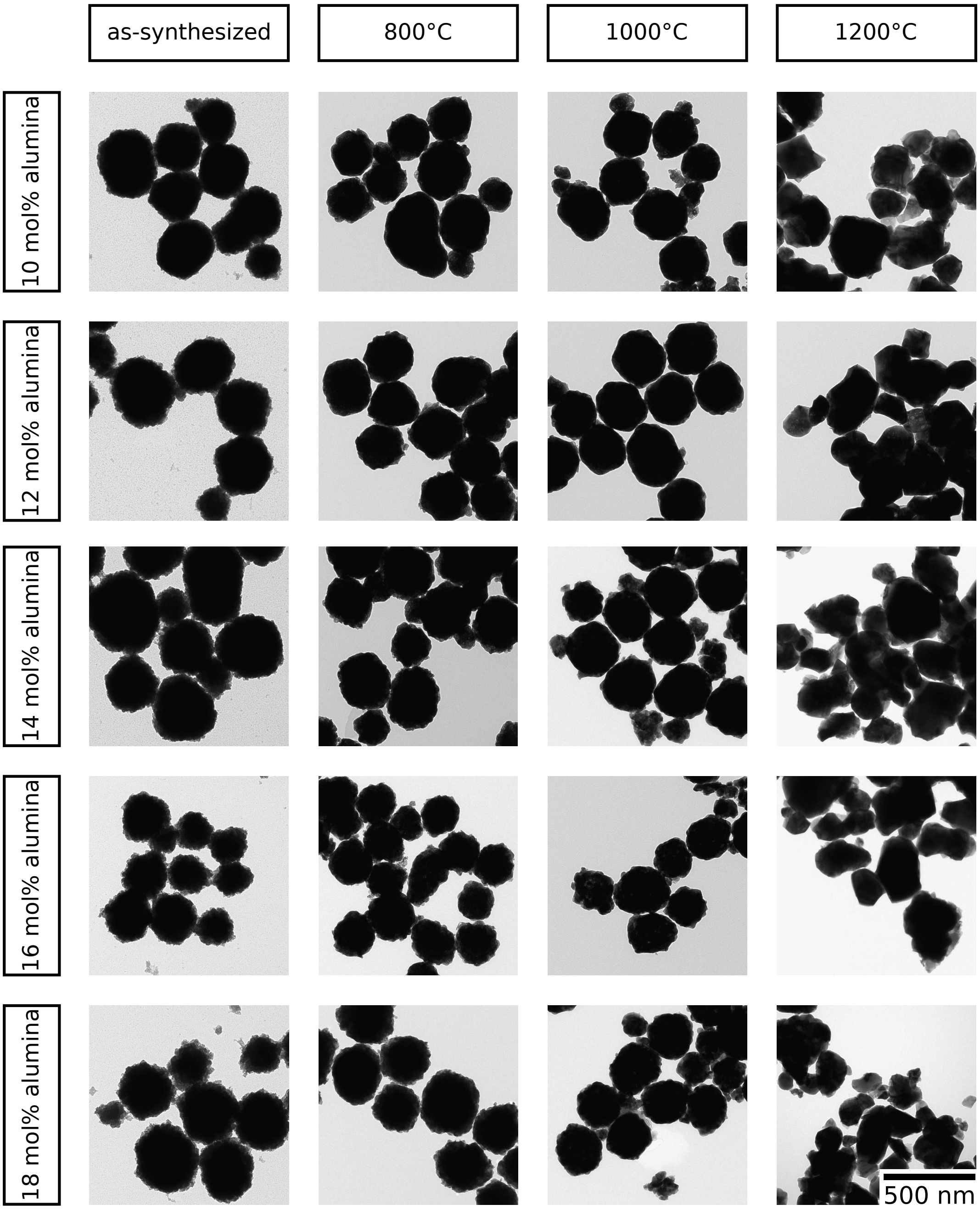

Figure S2. Representative TEM micrographs of zirconia particles doped with 10, 12, 14, 16, and 18 mol% alumina, as synthesized and after annealing at 800, 1000 and 1200 °C.

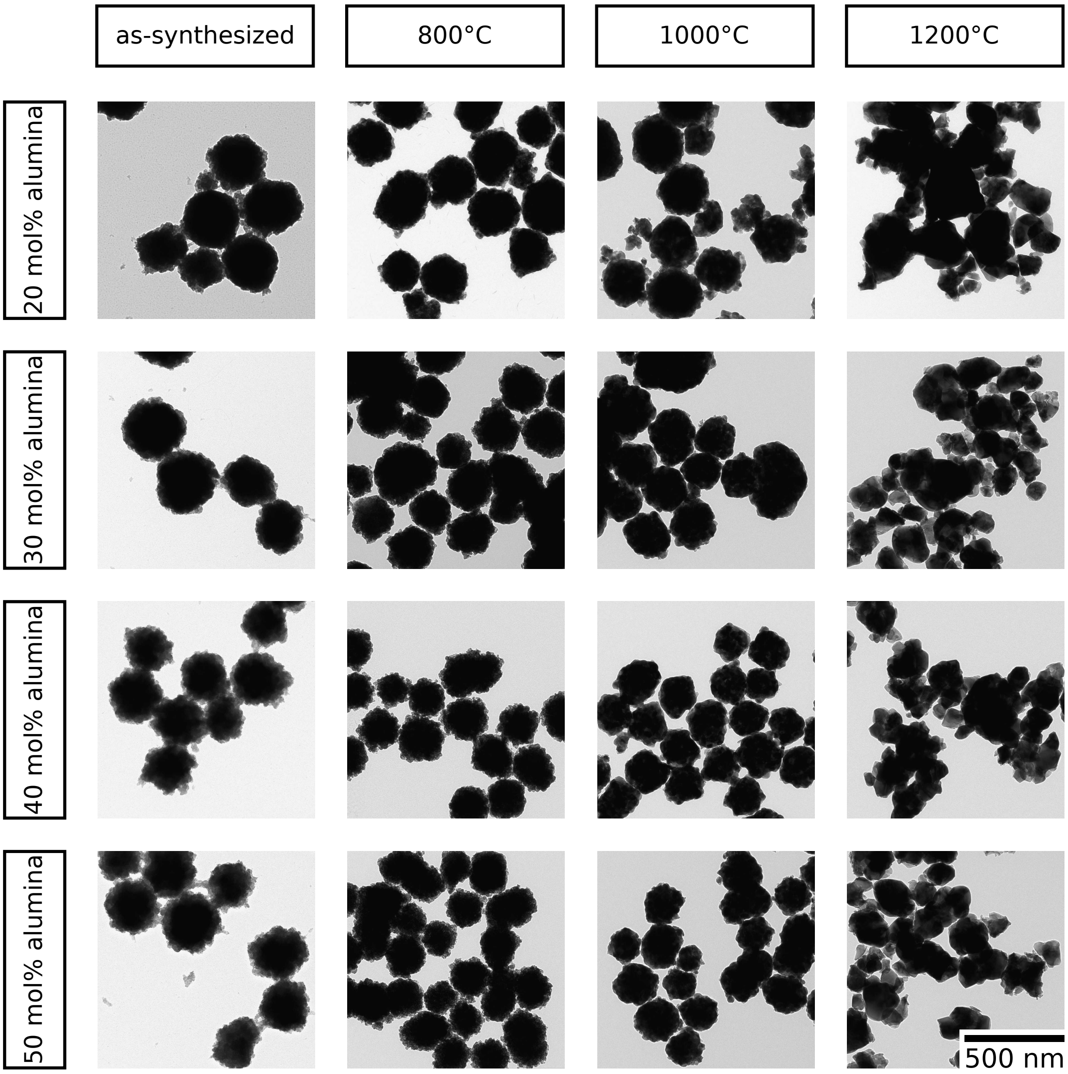

Figure S3. Representative TEM micrographs of zirconia particles doped with 20, 30, 40, and 50 mol% alumina, as synthesized and after annealing at 800, 1000 and 1200 °C.

Table S2. TEM, EDX, and ICP-OES characterization results for all as-synthesized particle samples. The theoretical alumina content corresponds to the employed precursor ratio Al/(Zr+Al). Mean diameters were obtained from the specified number of particles per sample and calculated assuming a perfectly circular cross-section. EDX data was obtained for individual particles, OES data are averaged values for a large number of particles. All errors indicate the standard deviation of multiple measurements.

	TEM		EDX	OES
theoretical alumina content/ mol%	number of particles	mean particle diameter/ nm	experimental alumina content/ mol%	
0	114	290.2 ± 30.6 (10.5%)	-	-
2	108	323.6 ± 36.3 (11.2%)	2.5 ± 0.38	1.86 ± 0.01
4	157	317.8 ± 42.4 (13.4%)	4.2 ± 0.31	4.34 ± 0.04
6	124	298.5 ± 28.8 (9.7%)	5.1 ± 0.48	5.76 ± 0.01
8	112	278.9 ± 32.0 (11.5%)	6.8 ± 0.49	8.53 ± 0.26
10	164	289.4 ± 33.4 (11.6%)	9.1 ± 0.31	11.00 ± 0.21
12	109	318.3 ± 32.6 (10.3%)	10.1 ± 0.68	12.28 ± 0.06
14	114	316.7 ± 49.2 (15.6%)	12.0 ± 0.39	14.82 ± 0.04
16	117	277.6 ± 40.0 (14.4%)	14.4 ± 0.09	16.48 ± 0.31
18	107	321.6 ± 39.6 (12.3%)	15.0 ± 0.47	18.69 ± 0.10
20	127	288.5 ± 40.7 (14.1%)	16.9 ± 0.22	21.35 ± 0.06
30	100	282.8 ± 40.5 (14.3%)	28.5 ± 0.51	35.11 ± 0.27
40	124	268.9 ± 23.7 (8.8%)	33.1 ± 0.47	40.31 ± 0.02
50	142	283.3 ± 28.9 (10.2%)	42.6 ± 0.52	49.67 ± 0.02

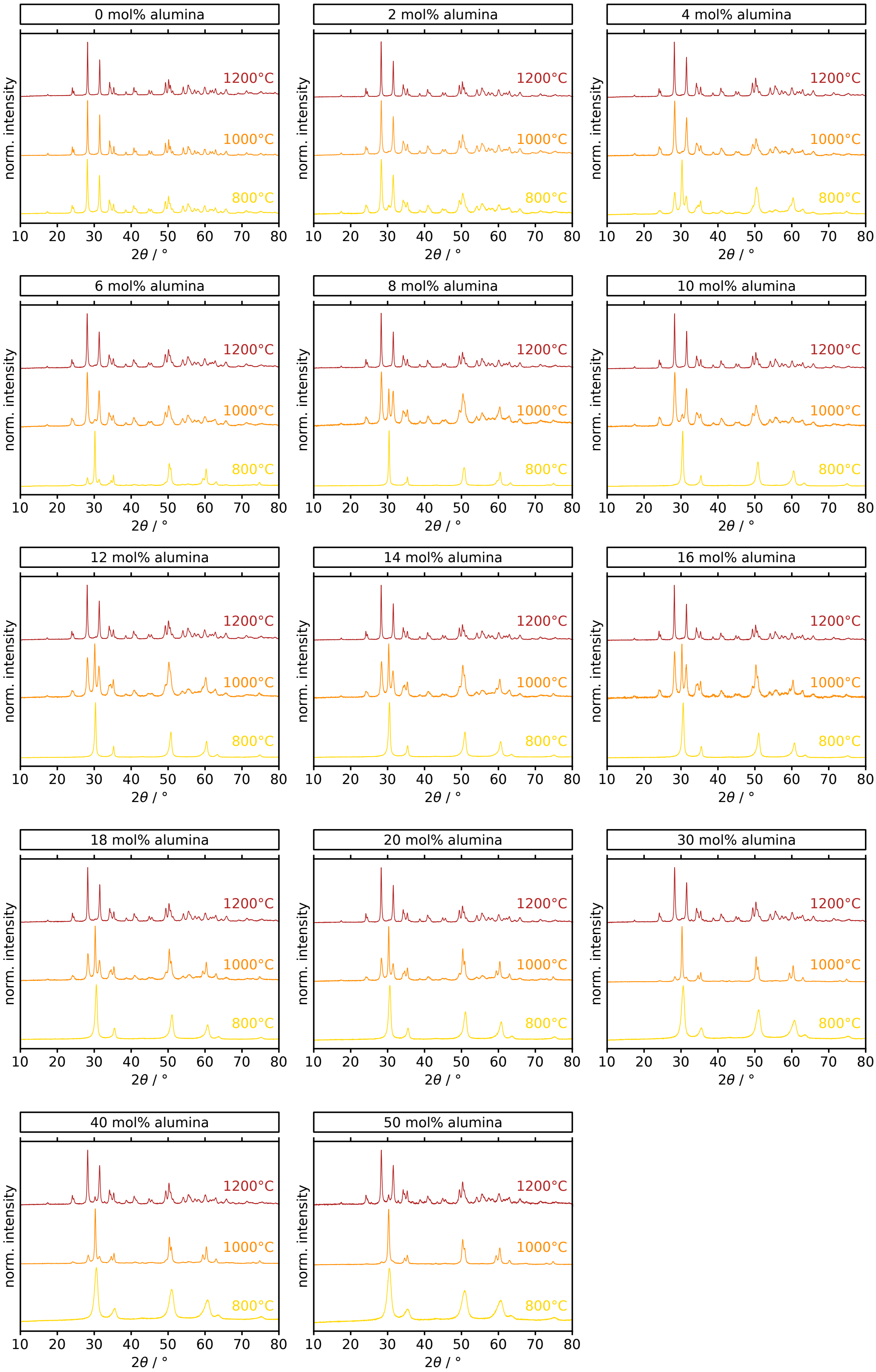

Figure S4. Powder X-ray diffractograms for all particle samples, after annealing at 800, 1000 and 1200 °C for 3 h.

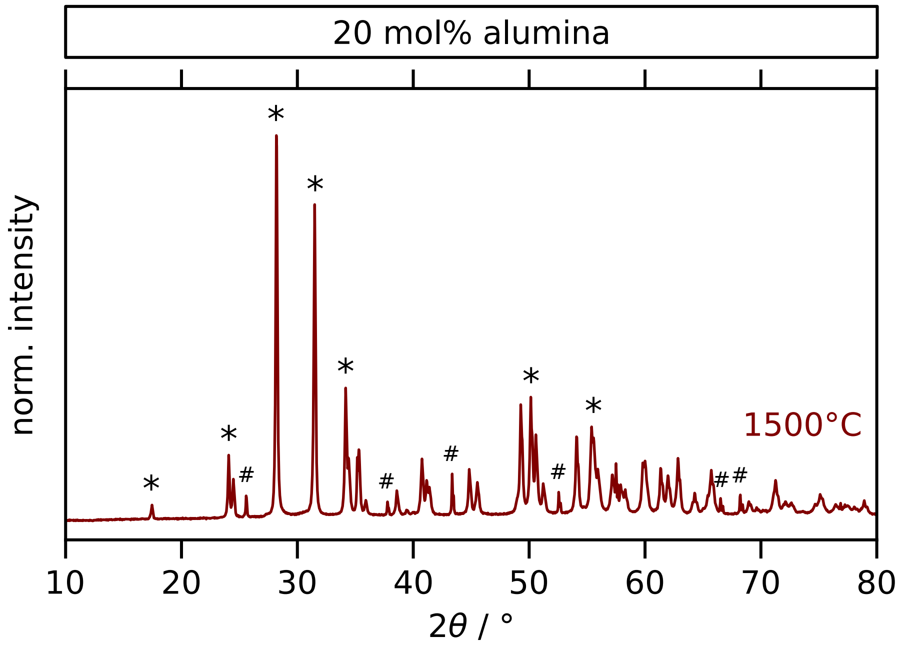

Figure S5. Powder X-ray diffractogram for a particle sample doped with 20 mol% alumina, after annealing at 1500 °C for 3 h. At this temperature crystallization of α-alumina (#) is clearly visible besides monoclinic zirconia (∗).

Table S3. XRD characterization results extracted from Rietveld-refined diffractograms for all particle samples, annealed at 800, 1000 and 1200 °C. $AlO_{1.5}$ denotes the alumina content, T the annealing temperature, pwf the crystal phase weight fraction, cs the average crystallite size, σ the goodness of fit, and R_{wp} the weighted profile R-factor of the refinement. The zirconia crystal phases are denotet by 'mon' for monoclinic and 'tet' for tetragonal. Errors represent the uncertainties in the refinement as given by the software MAUD. Values in brackets are considered inaccurate due to insufficient signal intensities.

$AlO_{1.5}$/ mol%	T/°C	pwf_{mon}/wt%	pwf_{tet}/wt%	cs_{mon}/nm	cs_{tet}/nm	σ/%	R_{wp}/%
0	800	97.06 ± 0	2.94 ± 0.2	44.171	(216.931)	3.93	8.21
	1000	98.95 ± 0	1.05 ± 0.12	69.944	(101.304)	4.62	9.63
	1200	99.31 ± 0.52	0.69 ± 0.07	99.683	(99.725)	4.14	8.33
2	800	83.22 ± 0	16.78 ± 1.34	31.123	17.187	3.14	7.76
	1000	98.75 ± 1.03	1.25 ± 0.12	62.253	(97.6)	4.44	8.78
	1200	99.08 ± 0.83	0.92 ± 0.09	85.952	(110.574)	4.91	10.83
4	800	49.89 ± 0.63	50.11 ± 0.39	42.856	26.266	2.91	6.35
	1000	72.61 ± 0.33	27.39 ± 1.63	35.175	143.682	3.32	8.67
	1200	99.11 ± 0.76	0.89 ± 0.07	75.415	(110.067)	4.28	8.56
6	800	38.07 ± 0	61.93 ± 1.66	22.444	46.917	3.65	8.76
	1000	72.99 ± 0	26.79 ± 0.52	35.707	92.377	3.82	8.03
	1200	98.62 ± 0.79	1.38 ± 0.08	71.407	(119.718)	4.08	8.30
8	800	8.77 ± 2.91	91.23 ± 0.98	(28.7584)	42.056	5.54	14.54
	1000	69.92 ± 0	30.07 ± 0.7	35.14	27.249	2.91	6.80
	1200	98.62 ± 0.77	1.38 ± 0.09	78.411	(89.388)	3.94	8.88
10	800	1.50 ± 0.21	98.50 ± 0.96	(6.529)	26.611	5.38	12.74
	1000	67.40 ± 0.43	32.60 ± 0.98	32.7	36.642	4.62	12.44
	1200	98.78 ± 0.75	1.22 ± 0.08	79.487	(120.177)	4.31	9.17
12	800	0.61 ± 0.27	99.39 ± 1.13	(46.95)	27.67	6.43	14.57
	1000	65.90 ± 0	33.79 ± 0.29	28.582	28.178	3.53	7.81
	1200	98.82 ± 0.71	1.18 ± 0.09	73.633	(116.135)	4.73	9.29
14	800	0.62 ± 0.13	99.38 ± 1.03	(30.373)	21.234	6.13	13.63
	1000	66.96 ± 0.74	33.04 ± 0.37	27.402	33.818	2.87	7.38
	1200	98.83 ± 0.88	1.17 ± 0.1	81.666	(96.814)	4.37	10.35
16	800	5.03 ± 0.86	94.97 ± 0.97	(43.277)	20.44	5.35	13.76
	1000	62.70 ± 0.64	37.30 ± 0.46	30.156	35.515	3.04	10.32
	1200	98.90 ± 0.86	1.10 ± 0.09	81.121	(122.794)	3.99	10.78
18	800	0.54 ± 0.18	99.46 ± 0.48	(98.942)	17.878	4.98	12.91
	1000	61.09 ± 0.5	38.91 ± 0.31	27.467	37.484	2.98	7.47
	1200	98.59 ± 0.86	1.41 ± 0.1	73.356	(73.31)	4.50	9.76
20	800	0.32 ± 0.14	99.67 ± 0.96	(97.286)	17.347	4.51	11.2
	1000	60.83 ± 0.34	39.17 ± 0.21	25.872	43.307	3.32	8.32
	1200	98.35 ± 0.71	1.65 ± 0.09	74.107	(118.225)	4.15	9.62
30	800	0.00 ± 0	100.00 ± 0.75	(100.0)	12.386	3.89	8.27
	1000	60.37 ± 0.77	39.63 ± 0.14	21.036	44.055	3.24	8.05
	1200	98.02 ± 0.9	1.98 ± 0.11	45.925	(126.672)	4.40	9.85
40	800	0.00 ± 0	100.00 ± 0.72	(100.0)	11.42	3.61	7.38
	1000	54.76 ± 1.08	45.24 ± 0.33	21.159	39.395	3.56	7.94
	1200	95.64 ± 0.74	4.36 ± 0.13	41.784	(43.177)	4.41	10.4
50	800	0.00 ± 0	100.00 ± 0.64	(70.375)	9.902	3.06	6.45
	1000	30.02 ± 1.1	69.98 ± 0.29	22.942	34.277	3.79	9.88
	1200	95.28 ± 1.06	4.72 ± 0.17	37.556	(133.199)	4.48	12.86

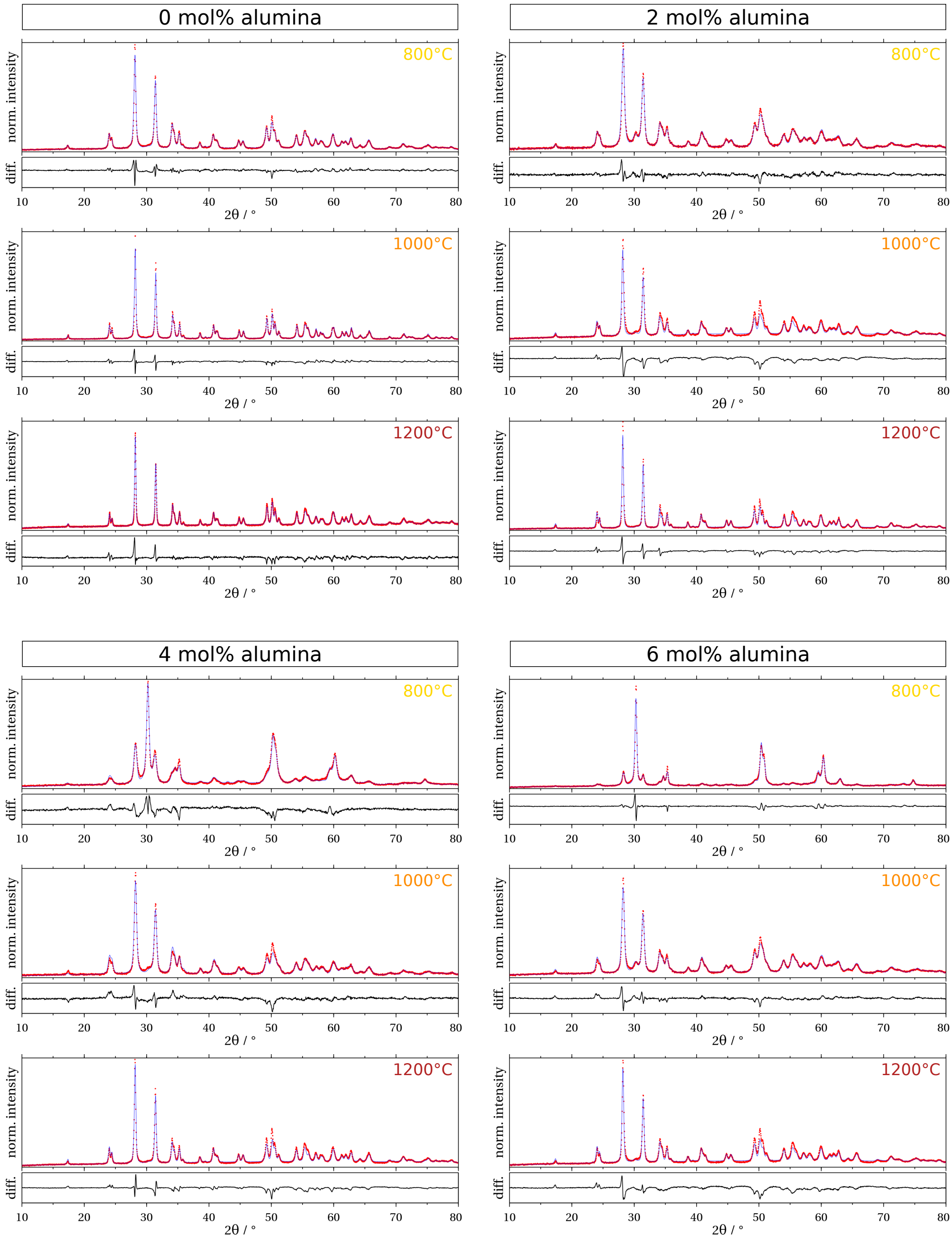

Figure S6. Rietveld graphs for particle samples with 0, 2, 4, and 6 mol-% alumina, annealed at 800, 1000 and 1200 °C. Expermiental data (red) was fitted (blue) using the Rietveld method after background correction. The difference (black) between experimental and calculated intensities is shown below the graphs.

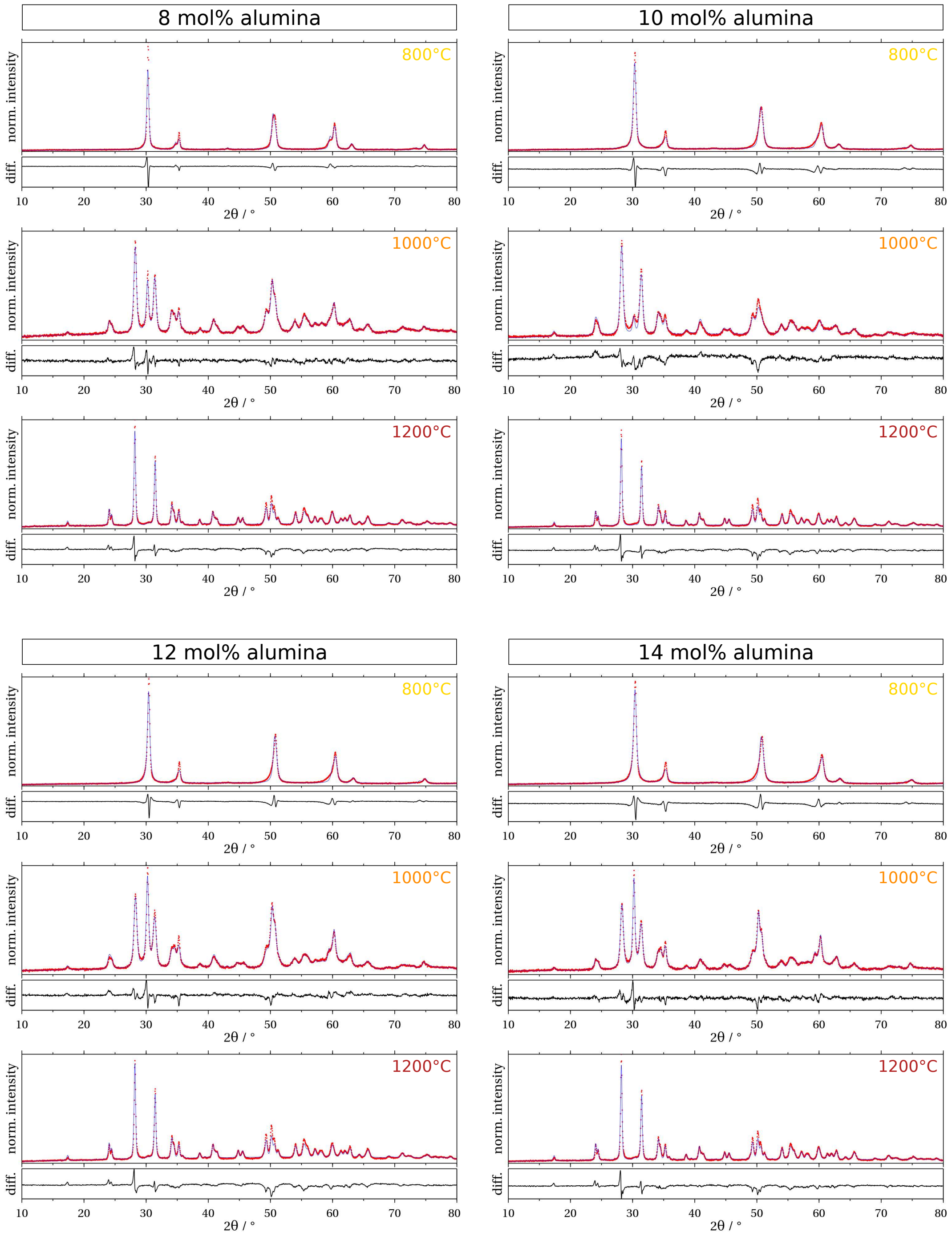

Figure S7. Rietveld graphs for particle samples with 8, 10, 12, and 14 mol-% alumina, annealed at 800, 1000 and 1200 °C. Expermiental data (red) was fitted (blue) using the Rietveld method after background correction. The difference (black) between experimental and calculated intensities is shown below the graphs.

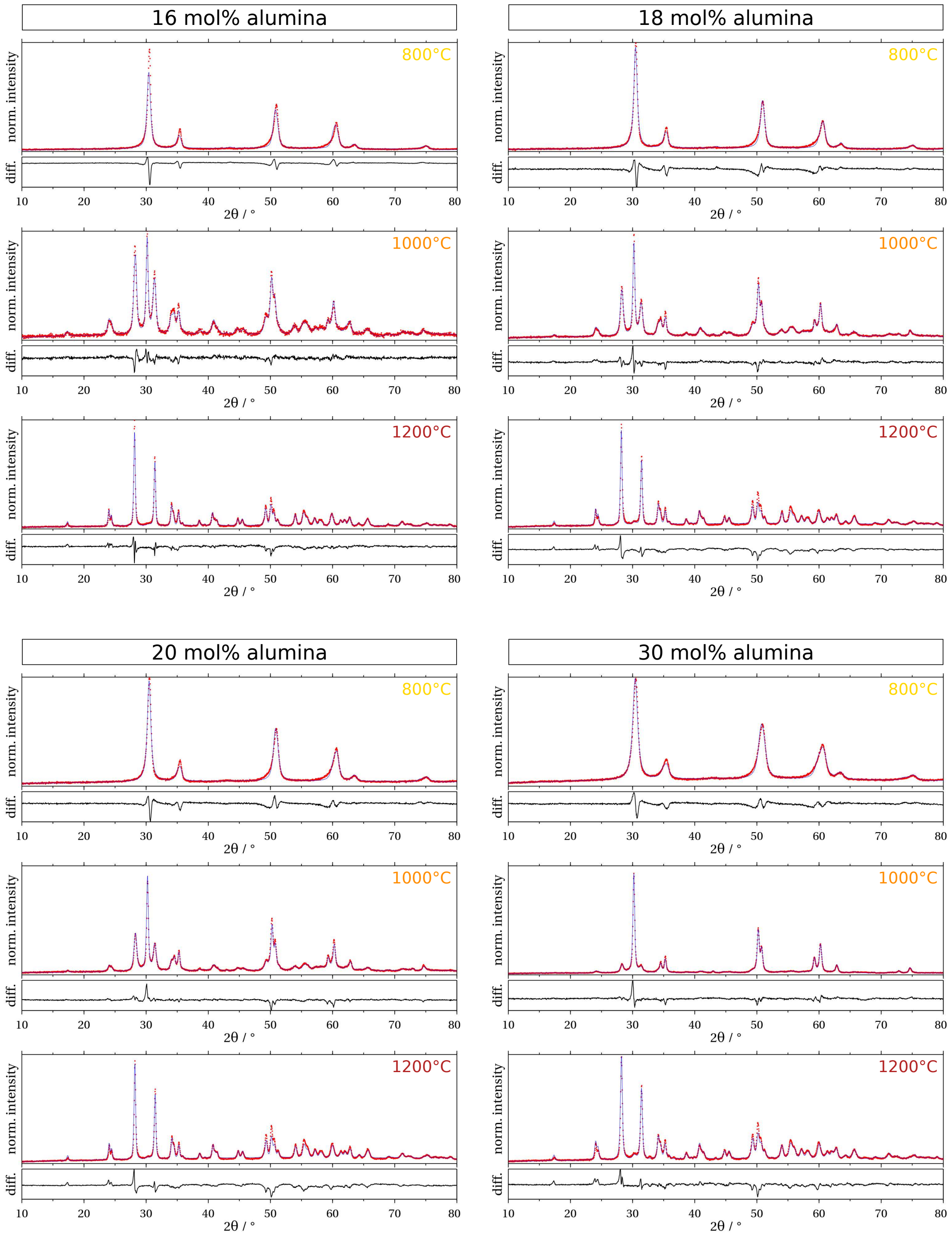

Figure S8. Rietveld graphs for particle samples with 16, 18, 20, and 30 mol-% alumina, annealed at 800, 1000 and 1200 °C. Expermiental data (red) was fitted (blue) using the Rietveld method after background correction. The difference (black) between experimental and calculated intensities is shown below the graphs.

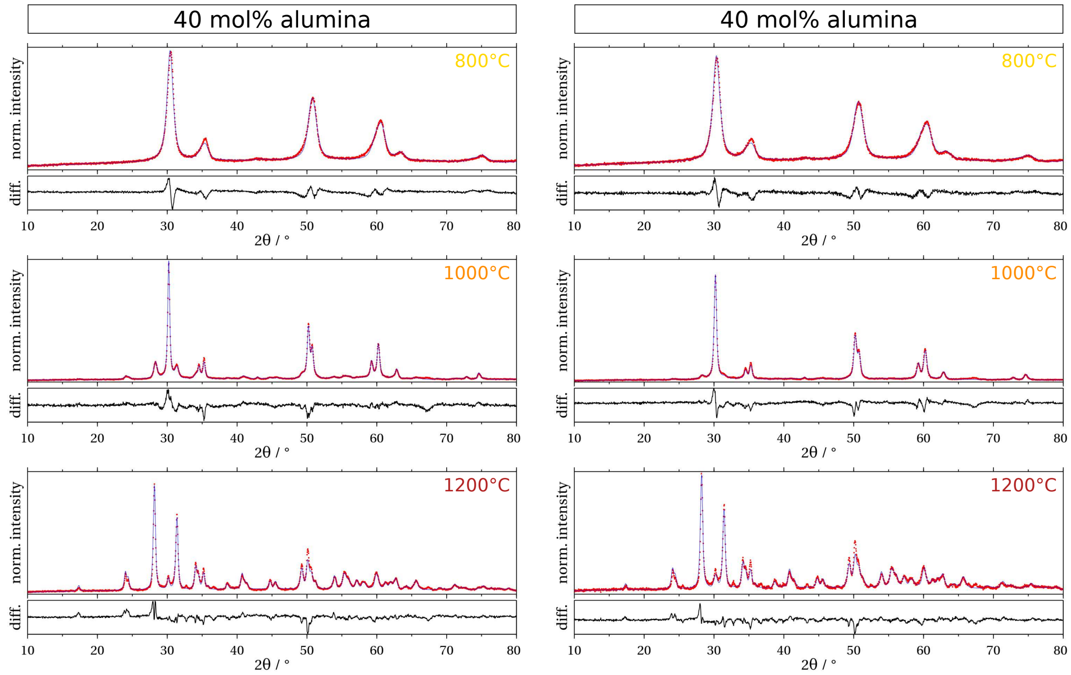

Figure S9. Rietveld graphs for particle samples with 40 and 50 mol-% alumina, annealed at 800, 1000 and 1200 °C. Expermiental data (red) was fitted (blue) using the Rietveld method after background correction. The difference (black) between experimental and calculated intensities is shown below the graphs.
